# Ten simple rules on how to set up an Innovation Boot Camp for exploitation of research results

**DOI:** 10.1371/journal.pcbi.1013807

**Published:** 2025-12-29

**Authors:** Susanne Hollmann, Christiane Hollmann, Lesley Tobin de Fuentes, Sady Roberto Rodriguez, Yerko Fredes, Antonio Garcia-Moyano, Christina Rørvik, Babette Regierer

**Affiliations:** 1 SB Science Management UG (haftungsbeschränkt), Berlin, Germany; 2 Potsdam University, Potsdam, Germany; 3 Rete Europea dell’Innovazione—REDINN, Pomezia, Italy; 4 Department of Chemical, Biological and Environmental Engineering, Universitat Autònoma de Barcelona, Bellaterra, Catalonia, Spain; 5 NORCE Research, Bergen, Norway; 6 Leibniz Institute of Vegetable and Ornamental Crops (IGZ) e.V., Großbeeren, Germany; Dassault Systemes BIOVIA, UNITED STATES OF AMERICA

## Abstract

In today’s research landscape, the dissemination and utilisation of results are not only an obligation but essential components of impactful research. Funding agencies are increasingly emphasising the translation of academic findings into practical applications that meet societal needs. However, researchers often lack training or incentives to focus on this task, leading to a disconnect between scientific enquiry and impact in practice. Innovation Boot Camps (IBCs) offer a dynamic solution to bridge this gap. IBCs provide a collaborative platform where researchers can familiarise themselves with attitudes and mindsets of end users, including industry professionals, policymakers and community members. This interaction ensures that scientific findings are aligned with real needs and challenges, increasing their relevance and applicability. These workshops foster creativity by encouraging researchers to think beyond the traditional academic framework and explore interdisciplinary approaches. This promotes not only innovative solutions, but also the effective tackling of complex societal problems. In addition to fostering creativity, IBCs also provide training to researchers in important professional skills such as communication, project management and business acumen. These skills are critical for communicating scientific concepts, navigating commercial environments and successfully implementing research findings. By participating in IBCs, researchers can assume a proactive role in transforming their findings into marketable or socially relevant innovations. This article offers a step-by-step guide on how to structure and run an effective Innovation Boot Camp, helping researchers transform their results from bureaucratic obligations into strategic opportunities for societal benefit.

## Introduction

For funders and research institutions, the transfer of research findings into commercial and societal applications is becoming increasingly important. Funding organisations identified the need for specific training decades ago and established training frameworks like the U.S. National Science Foundation Innovation Corps [1] and the European Commission’s Joint Research Centre EntreComp [2]. However, transfer activities are not yet an integral part of daily research processes and may present significant challenges for scientists. Bridging the gap between academia and industry requires an interdisciplinary approach, integrating perspectives from different fields to tackle complex problems in an innovative way.

Innovation Boot Camps (IBCs) offer a structured, hands-on format to guide researchers in this transition. These dynamic workshops, spanning 2–5 days, start with predefined challenges or business ideas, guiding attendees from initial exploration to concrete concepts, product or service prototypes. Along the way IBCs help to identify potential customers and key implementation steps.

During IBCs, attendees can develop prototypes, refine ideas based on stakeholder feedback, and draft business plans. The underlying iterative process ensures that innovations are scientifically sound, economically viable, and socially relevant. The inclusion of non-experts, such as end users and industry experts, can bring fresh perspectives that challenge assumptions and enhance usability and effectiveness. This co-creation approach democratises innovation and encourages acceptance and adoption in the real world. Moreover, participation in IBCs offers researchers the opportunity to develop important professional skills, such as project management, stakeholder engagement, business concept development and effective communication. These skills are crucial for securing funding, collaborating with industry and translating academic work into marketable innovations. In addition, IBCs improve the visibility and credibility of researchers and open doors to new funding, collaborations and career opportunities.

Beyond individual skill-building, IBCs redefine the dissemination of research results as a strategic opportunity for societal impact. By linking academic knowledge with practical applications, these workshops ensure that research is not just theoretically sound, but also transformative helping to address challenges in technology, healthcare, sustainability, and beyond.

Organising an IBC for novices requires careful planning to ensure it remains engaging, collaborative and productive.

**Fig 1:**
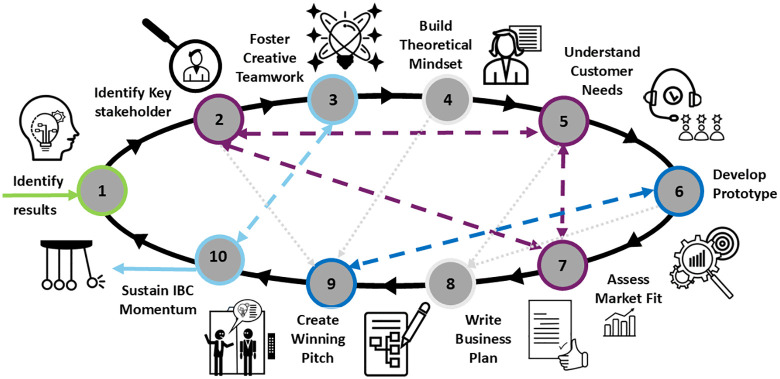
Workflow Innovation Boot Camp. The flowchart demonstrates an optimal workflow for conducting an Innovation Boot Camp for the Exploitation of Research Results. The arrows demonstrate the iterative or reciprocal relationships between the individual rules.

This paper describes “Ten simple rules on how to structure and run an effective IBC for your applied research project”. Each rule offers concrete, actionable measures to ensure the IBC is both goal-driven and responsive to participant experiences, fostering meaningful engagement and impactful outcomes (Fig 1).

## Results

### Rule 1: Identify results suitable for exploitation and clarify the IP boundaries

Begin by identifying the test case for the potential innovation (product, service, or process) you will address. This case serves as the basis for all subsequent IBC activities, including customer identification, stakeholder analysis and market assessment. The test case should describe the core innovation, the problem it addresses and the desired outcomes. In the IBC cohort on which this paper is based, participants applied computational biology approaches to develop a sustainable, enzyme-based sunscreen. For target identification, they used sequence analysis, structural modelling, and active-site prediction to select candidate enzymes, such as laccases and lipases, which may provide UV-protective activity and enhance sunscreen formulation stability. During lead optimisation, molecular docking and molecular dynamics simulations were applied to predict enzyme structural stability and substrate–active site binding affinity, allowing the team to prioritise the most promising variants for experimental validation. By integrating these computational insights, the cohort aligned their research approach with their start-up vision, focussing resources on high-potential candidates and improving the likelihood of successfully developing a bio-based, sustainable product. To enhance understanding and illustrate how the tools can be applied beyond the main test case; the IBC may introduce additional fictional examples. One such example is a screening platform to detect pharmaceutical residues in water. In this scenario, participants would define the nature of the innovation (e.g., a rapid, cost-effective biosensor for trace contaminants), the public health or environmental issue it addresses, and the anticipated benefits (e.g., improved monitoring, early detection, or regulatory compliance). While our fictional example is drawn from environmental monitoring, similar frameworks apply to computational biology (e.g., diagnostic tools, algorithm-based health risk assessments).

Whether working on a real or fictional test case, teams should clearly articulate the type of innovation (product, service, or process), the technical or societal problem it seeks to solve, the primary users, and the intended impact. Clarifying these elements early on ensures coherence throughout the IBC and equips teams to communicate their innovation credibly to stakeholders (Rule 2), users (Rule 5), and potential markets (Rule 7).

Once you have established your test case, attend to any Intellectual Property (IP) considerations. Begin by determining the ownership of the technology, know-how, and materials involved. If your project is part of a collaborative effort, such as an international research initiative, notify your Project Coordinator and partners about your plans for the test case. Identifying all the contributors to the technology is essential to prevent conflicts or violation of rights that could restrict its use in the IBC. If primary IP responsibilities are unclear, consult your institution’s Technology Transfer Office (TTO) for guidance.

Clarify everyone’s interests and secure written permissions to share information with IBC attendees. Also, ensure you have “freedom to operate” by conducting due diligence on existing patents and publications to verify that your idea does not infringe on others’ rights. Revisit this legal landscape after the IBC and during further sensitive information development to pre-empt any legal complications.

Confidentiality is paramount for IBC attendees. If proprietary details are to be disclosed, use a Non-Disclosure Agreement (NDA) [3] to protect such information. An NDA defines what is confidential and binds attendees to privacy. Ensure attendees understand the legal implications of breaching an NDA as this can lead to legal consequences, including financial penalties.

In summary, a successful IBC relies on a clearly defined challenge, rigorous IP and legal checks, and the incorporation of protective measures like NDAs to safeguard both the initial invention, IP, and the innovative solutions developed within the IBC.

### Rule 2: Understand key stakeholders: Enablers, influencers and barriers

Once the test case has been defined, IBC participants must consider a broader network of actors beyond direct users: stakeholders. A stakeholder is ‘any group or individual that can influence or be influenced by the achievement of an organisation’s goal’ [4]. This can include regulatory authorities, suppliers, investors, interest groups, distributors, retailers, competitors and others.

Stakeholders can play various roles—enablers, blockers, mentors, or amplifiers—depending on their position and influence. IBC attendees must therefore think beyond the typical frameworks of their research community and consider broader applications and translational pathways. This is particularly critical in computational biology-focussed IBC programmes, where research insights are expected to evolve into data-driven platforms, predictive tools, or therapeutic innovations.

For example, in our computational biology test case involving sequence analysis, structural modelling, and active-site prediction to identify candidate enzymes for enhancing UV-protective activity and sunscreen formulation stability, stakeholders include bioinformatics software providers, enzyme manufacturers, cosmetic formulation partners, regulatory agencies overseeing product safety, and investors focussed on sustainable biotechnology. Each of these actors influences the feasibility, regulatory compliance, and eventual adoption of the innovation. Academic collaborators and contract research organisations specialising in enzyme expression and testing are also key enablers who bridge the gap between computational predictions and real-world formulation testing.

Similarly, in our fictive screening platform to detect pharmaceutical residues in water, stakeholders include environmental monitoring agencies, municipal water suppliers, biosensor developers, public health authorities, and TTOs. In this scenario, participants define the nature of the innovation (e.g., a rapid, cost-effective biosensor for trace contaminants), the public health and environmental challenges it addresses, and the anticipated benefits for regulators, communities, and industrial users. Data scientists and bioinformatics experts involved in signal interpretation and predictive modelling form an essential stakeholder group, ensuring that the computational algorithms driving detection are accurate, transparent, robust, and adaptable to emerging pollutants.

Across both cases, stakeholders provide access to datasets, validation resources, regulatory guidance, and market insights—all of which are critical for translating computational biology innovations into viable, socially beneficial products.

Conversely, stakeholders could also be competitors or regulators and thus may have a negative influence on the development of the innovation. Knowing and onboarding these stakeholders might be useful, but be aware of the IP boundaries set in **Rule 1**. Ideally, representatives of the main stakeholder groups will participate in the IBC, thus allowing their insights to be integrated from the outset [5].

After identifying stakeholder groups, assign potential benefits to each listed group in relation to your innovation. Benefits may be direct or indirect: for example, children may benefit from their parents applying a new brand of sun protection. Creating a map of stakeholders can enable the latter assignment of specific roles within the IBC to these groups, such as sponsor (**Rule 9**), advisor, tester, interviewer, or personas for persona modelling (PM) (**Rule 5**). It should be noted here that the group that stands to benefit most from the product may even become advocates for your project and, ideally, could be engaged as mentors or investors—not only for the duration of the IBC but also in the ongoing development of your innovation.

Importantly, stakeholders must be clearly distinguished from users (Rule 5): while users directly interact with the product, stakeholders influence the context in which it operates. Teams must also remain aware of IP boundaries and confidentiality requirements (see Rule 1). Mapping stakeholders early allows IBC attendees to build a coalition of support while preparing for systemic resistance or competitive risks.

Identifying and mapping stakeholders helps IBC teams recognise enablers, blockers and beneficiaries of innovation—laying the groundwork for strategic engagement and aligning with user and market insights developed in Rules 5 and 7.

### Rule 3: Create an environment of openness and creativity to foster ‘out of the box’ thinking and teamwork

To organise a successful IBC, planning and coordination should ideally begin not later than three months in advance. This process starts with forming a core team of at least two key members: the organiser, responsible for logistics and planning, and a representative who is knowledgeable about the product or test case that is driving the IBC. The optimal core team will comprise individuals who not only bring diverse expertise but also foster an environment of openness and creativity to encourage ‘out of the box’ thinking. The core team will also be responsible for preparing the necessary information material and handouts. When core team members hold multiple roles, clearly defined responsibilities help ensure smooth collaboration from planning through to follow-up. As the IBC structure takes shape, the core team will design a concise, high-impact schedule with well-defined time blocks and objectives to keep the event focussed and engaging. In addition, a strategy for documenting results needs to be planned and prepared.

Importantly, the IBC facilitating personnel should include individuals who are specifically skilled at evoking innovative ideas, identifying project outcomes with exploitation potential, and addressing IP considerations; a local organiser adept at effectively managing on-site requirements should also be recruited. Experienced facilitators are required to complement the core team and to provide foundational knowledge, introduce attendees to IBC principles, and actively support the teams, helping navigate technical challenges and encourage dynamic discussions.

A suitable location with space and flexible facilities is essential for unlocking creative potential. Through the well-established creativity technique of ‘Design Thinking’, and practical crafting, painting, playing and experimenting with different materials, empathy and creativity are fostered, which are conducive to achieving more effective and human-centred solutions. This Design Thinking approach breaks down barriers and hierarchies as well as creating a supportive environment in which ideas can flourish. Ultimately, this structured yet dynamic process not only accelerates the understanding and development of solutions but also establishes a sense of ownership and acceptance among all attendees, making an IBC both enjoyable and effective for all.

Participant selection is another fundamental step towards success. Depending on whether test cases are customer-related or consumer-focussed, the core team may comprise attendees with relevant backgrounds and different disciplines to enable the holistic inclusion of multiple perspectives (**Rule 2**). For example, the team should involve a computational biologist to explain enzyme modelling, a cosmetic formulation expert to assess UV-protective potential, and a sustainability-focussed participant to ensure environmental compatibility. Moderators guide the team in applying creative problem-solving directly to the biological and formulation challenges, ensuring that computational insights are effectively translated into actionable prototype concepts. This targeted selection enhances engagement by aligning attendees’ expertise with the IBC’s objectives. Small teams of four to six members work best, as this facilitates more focussed collaboration and the fostering of a diversity of ideas. Each team has its own facilitator to maintain momentum, keep tasks on schedule, and support team dynamics.

A successful IBC requires early planning, a well-structured core team, skilled facilitators, a creative and flexible environment, and carefully selected attendees, all working together to foster innovation, collaboration, and impactful solutions.

### Rule 4: Establishing the mind set—Build up the theoretical background

To kick off the IBC, the attendees will require an introduction to the concepts and tools that will be used during the workshop. In the IBC plan, incorporate sessions to introduce the theoretical background for the practical work. Our recommendation is to deliver a dedicated session prior to the specific exercise to avoid overwhelming attendees with theory. This part of the IBC programme will combine and deliver the theoretical elements of Design Thinking [6] and Business Plan development [7,8].

The Design Thinking method [9,10] provides with the foundational knowledge to effectively use tools for exploring, iterating, and achieving impactful outcomes (aligned with Rule 5). These creativity methods, central to the IBC, enable attendees to transform abstract challenges into tangible, user-focussed innovations with the potential to create meaningful change [11]. A brief theoretical introduction helps non-computational biologists understand how sequence analysis and structural modelling influence the selection of enzyme candidates for UV protection. This could be supplemented by a short exercise or demonstration showing how enzyme variants influence the stability of sunscreens, or by simplified molecular visualisations illustrating how interactions at active sites influence UV absorption. By directly linking design thinking principles to the computational biology workflow, participants gain both a conceptual and practical understanding of how theoretical models are translated into real-world innovations.

Following the Design Thinking process, tools for the creation of a business plan are introduced to push further development. A business plan is the blueprint for companies and is key to attracting investors and securing funding. The attendees will require an overview of the various components and aspects of a business plan outlining the business’s objectives, strategies, and operational procedures, with a clear focus on targeting internal and external stakeholders, investors, and other key entities. The later exercises (**Rule 8**) will build on this groundwork and use the Business Model Canvas [12]. This is a high-level overview that helps to quickly test and iterate the business ideas and their proposed implementation (**Rules 9 and 10**).

A well-structured IBC begins by introducing attendees to key concepts and tools, combining design thinking for creative problem solving with business plan development to refine and validate ideas, ensuring a user-centred, strategic approach to innovation.

### Rule 5: Know your customer needs: Empathy before design

**Fig 2:**
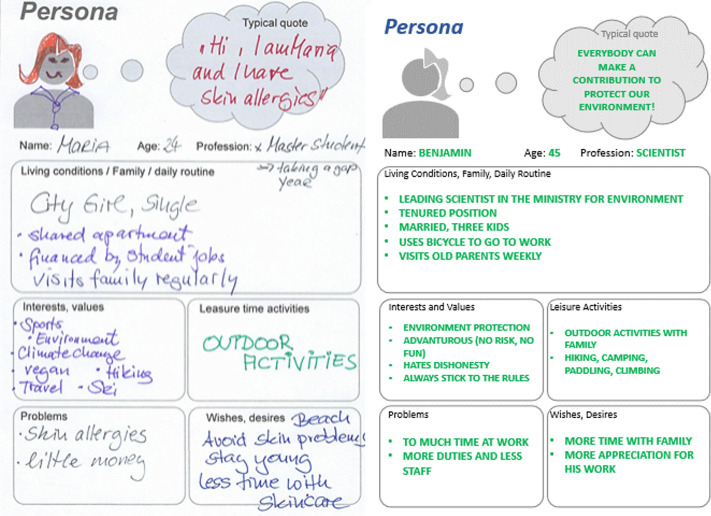
Persona modelling: two user archetypes. The use of ‘personas’ as archetypes of the users of an innovation helps to understand the problems, motivations, needs and emotions of potential users and to identify commonalities in terms of purchasing preferences, social relationships, consumption habits and age. Two personas are shown here: Maria, developed during our real-life IBC event to represent an end user in the case of the sunscreen innovation, and Benjamin, a fictional decision-maker persona for a screening platform for detecting drug residues in water. Together, these examples illustrate the importance of capturing both the user and decision-maker perspectives to support user-centred innovation design.

Understanding user needs is fundamental to the success of a product. Before beginning product design, IBC teams must develop a deep, evidence-based understanding of their target users—their needs, behaviours, and contexts of use. This ensures that innovations address real challenges, not just perceived problems. A key method for developing user insights is PM [13]. Personas are fictional representations of user archetypes based on research findings. They help to identify motivations, needs, weaknesses and behaviour patterns. During the IBC, teams are expected to create at least one persona per test case. These should reflect a range of user groups—e.g., families, pensioners, athletes or allergy sufferers for the sunscreen test case—taking into account factors such as age, gender, income, education, religion and occupation [14,15]. This approach enables the development of customised solutions and useful features. Ultimately this helps prevent the common mistake of designing a product based on assumptions. The teams should then agree on one or two personas and describe their characteristics and needs using templates and role play. In our example, the developed persona is Maria, a 25-year-old student from Spain. She finances herself through student-type jobs and thus lives on a limited budget. She has a significant interest in outdoor sports and environmental issues (Fig 2). While end-user personas like Maria provide deep empathy into product usage, it’s equally important to identify decision-maker personas—those who authorise or influence purchasing decisions. For example, children are the end-users of sunscreen, but parents are the decision-makers who purchase and apply it. Or, for our fictional case of a screening platform, Benjamin is a 45-year-old leading scientist at the Ministry for Environment. He holds a tenured position, is married, has three children, cycles to work, and spends weekends hiking and camping with his family. He is rule-abiding, values honesty, and sees environmental protection as a personal and professional mission, and he may decide whether the solution is adopted, even he is not the direct user. Both perspectives are critical to understand and validate. Mapping both personas ensure the innovation meets functional and strategic expectations.

Once the personas are defined, the attendees can then map out their ‘journey’ with the product to understand its practical applications and identify required features. This Customer Journey Mapping (CJM) [16] illustrates how, when and why a persona interacts with the product, revealing their experiences, pain points and motivations. This process can also highlight any problems with the original idea, and the need to redefine challenges. Teams describe interactions and touchpoints using imagery and narrative to encourage immersion and creativity. Providing tools such as paper and pens encourages hands-on exploration in the exercise, making the process dynamic and engaging.

**Fig 3:**
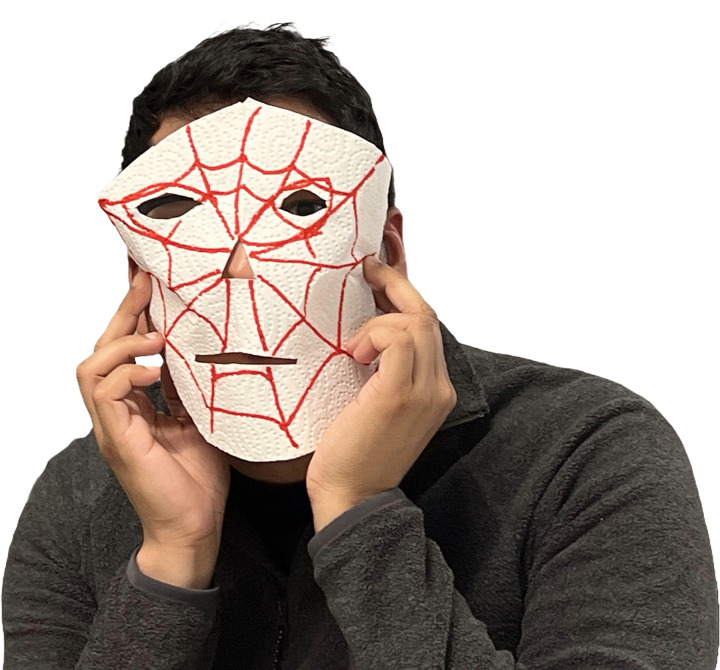
Example of prototyping through tinkering. In our scenario, a prototype of a sun protection mask made of paper was developed to showcase the potential product.

To complement PM and CJM, teams may incorporate short, anonymous user surveys, either via online forms or street interviews [17]. To ensure relevance, surveys should target users’ most pressing pain points—what frustrates them about current solutions, what remains unmet, or what would compel them to switch—while also helping to validate personas and uncover key data such as usage frequency, price expectations, and product preferences. The findings from this process are carefully documented to serve as a foundation for Business Plan development (**Rule 8**). It is important to set a clear time limit for this process. Clearly define time constraints—e.g., 15 min to develop questions, and 45 min for data collection in a street survey (Fig 3). Ensure General Data Protection Regulation [18] compliance, and, where applicable, obtain permissions for conducting street interviews from local authorities. By combining PM, Journey Mapping and user surveys, IBC attendees gain actionable insight for user-centric innovation and practical prototyping.

Understanding users through persona and journey mapping ensures that innovations address real needs—forming the basis for stakeholder relevance (Rule 2) and targeted market positioning (Rule 7).

Customer discovery is not a linear process. As teams gather insights from users (Rule 5), engage stakeholders (Rule 2), and assess market dynamics (Rule 7), they may uncover previously unrecognised pain points, barriers or new opportunities. This can—and should—lead to pivoting the original concept, adjusting the product’s value proposition, or redefining the target segment. Encouraging such flexibility during the IBC prepares teams for real-world innovation, where adaptation is often key to success.

### Rule 6: Expand to practice—Develop a prototype

The most enjoyable phase of IBC is when attendees translate their idea into a tangible product or service. These ‘prototypes’ do not have to be perfect, but they should be able to convey important features and functions or solve a particular problem. Prototyping is an essential step in the IBC process for quickly iterating ideas, gathering valuable user insights, minimising risk, and ensuring the final product is well-aligned with user needs (**Rule 5**). First ask the IBC attendees to identify the specific concept or feature of their business idea that they want to prototype. This could be a user interface, a product design, or a specific functionality. In a purely computational biology context, this might also mean visualising or demonstrating how a data-driven workflow, model, or analysis step would function in practice—for example, through a flowchart of a bioinformatics pipeline or a mock-up of a data interpretation interface.

Attendees choose a prototype form that lends itself to their testing needs. For example, a low-fidelity prototype such as hand-drawn sketch or wireframe is ideal if they are working on a digital product, or they can create simple, tangible models using available materials for a physical product. Projects in the field of computational biology benefit from rapid, tangible visualisation—for example, by sketching the workflow from sequence analysis to structure prediction, or by using colour-coded maps to represent data sets, algorithms and results, as these exercises help to clarify the logic of innovation and the roles of the stakeholders involved. Ensure you have all the necessary materials. Gather crafting supplies such as paper, pens, scissors, glue, sticky notes, junk (boxes and bottles) and, where appropriate, laptops, digital tools, software modules or flowchart software to visualise data flows or demonstrate key steps. These materials will enable attendees to quickly create different prototype models, from simple sketches to more elaborate physical forms. Digital tools such as wireframing software or 3D printing resources may also be used depending on your project needs.

Once you have created your prototype, it is time to test it. Engage attendees—team members, potential users, or other stakeholders—to interact with the prototype. In our case, the teams decided to create a sunscreen application mask for children who like superheroes (Fig 3).

During this engagement, it is important to gather feedback so that you can further refine the prototype based on these insights. This may involve adjusting features, simplifying designs, or addressing issues that users faced during testing. You can repeat this iteration process as many times as needed, gradually improving the prototype until it meets user expectations. The teams’ moderators should monitor this process to ensure that the prototype effectively addresses the core problem that it is designed to solve and aligns with both user needs and business goals. The prototype also serves as a visual aid in communicating ideas to stakeholders later (**Rule 9**). Thus, at this point it is important to save the prototypes for subsequent exercises within the IBC.

The prototyping phase is the most engaging part of the IBC, where attendees transform abstract ideas into tangible solutions, using low-fidelity or physical models to test concepts, gather feedback, refine designs, and ensure alignment with user needs and business goals. Furthermore, this process combines digital and physical approaches and helps participants explain complex workflows, validate assumptions, and visualise and understand abstract, data-driven ideas in a collaborative workshop environment.

### Rule 7: Know the market: Assess fit and survival probability of your prototype

User needs are crucial, but a profitable market is equally important. To assess whether a prototype can be successful beyond the IBC environment, participants must conduct a basic market fit and feasibility analysis. While in-depth strategic planning is beyond the scope of the IBC, attendees should develop a foundational understanding of market dynamics and competitive positioning.

This can be achieved by guiding the teams through three core areas of analysis: industry type, market segmentation, and competition. Depending on the available time, these can be addressed through short 30–45 min or in-depth exercises.

Industry Type: Teams begin by identifying the sector relevant to their product (e.g., cosmetics, health tech, environmental analysis assets) and assessing its overall characteristics. This includes researching factors such as market growth potential, innovation trends, regulatory challenges, and competitive dynamics. Each team member can focus on a specific aspect, with findings discussed collectively. A 30-min research period is usually sufficient for this activity. This step helps teams assess whether the industry can support their product’s entry and growth [19].

Next, participants segment the market based on user types, use cases, or value propositions (Market Segmentation). For example, the sunscreen market could be divided into segments like everyday users, sports enthusiasts, or consumers seeking eco-friendly or allergen-free products. For the screening platform designed to detect pharmaceutical residues in water, the market can be divided into segments like research institutes and universities, private and testing laboratories, waste water producers, environmental monitoring agencies and municipal authorities. By clearly identifying and prioritising these segments, IBC participants can align product development with the most promising and receptive customer groups, enabling targeted marketing strategies and tailored feature sets. This step builds directly on the persona models developed in Rule 5 [13,14], helping teams define their target audience and identify where their product will have the greatest relevance and appeal. Allocate approximately 30 min for this exercise, using criteria such as demographics, location, usage patterns, and consumer needs [15].

The final stage is a competitor analysis. The teams examine direct competitors (e.g., similar product brands) and alternatives (e.g., portable UV protection products, skin care substitutes, or waste prevention systems), as well as existing enzyme research platforms or biosensor technologies, to understand how their prototype compares in terms of performance, cost, or ease of use. This can be done through online research or in case of the sunscreen test case by short field visits to nearby stores. Attendees evaluate the competition’s strengths, weaknesses, customer base, and pricing strategies to identify market gaps and differentiation opportunities.

These insights feed into a comprehensive SWOT analysis [19], which helps teams assess internal strengths (e.g., sustainability, cost-efficiency), weaknesses (e.g., low brand awareness), opportunities (e.g., rising demand for green products), and threats (e.g., market saturation, regulatory risk) [20]. A 45- to 60-min time slot is recommended for this final analysis phase.

By the end of this process, attendees will have identified the unique value proposition of their prototype and outlined key strategic considerations for market entry. A well-structured market assessment equips IBC teams with the fundamentals of industry insight, audience targeting, and competitive positioning, enabling them to refine their product’s viability and craft strategies to navigate challenges and seize opportunities. Market viability depends on aligning product features with user needs (Rule 5) and the expectations of key stakeholders (Rule 2), enabling strategic segmentation and competitive advantage. This exercise helps participants to position their prototypes realistically—by assessing where their innovations offer added value, gaining initial partners and contributing to long-term translational impact beyond the IBC context.

### Rule 8: Implement your prototype: Develop business plan

The next logical step in your IBC is to create a business plan that incorporates all the findings and results of Rules 1–7. A business plan serves as a strategic roadmap that captures the key considerations for realising a business idea. The process of developing and refining the plan creates clarity in the business, especially when it comes to raising capital beyond one’s own savings. A well-developed plan demonstrates a solid understanding of the business and its vision and helps to prioritise the most important tasks [21]. Before performing the exercise of a Business Model Canvas (BMC) [22], ask the teams to reflect and integrate the information they have received from Rules 1 to 7 and then answer the following questions presented in Table 1.

**Table 1:** Questions to identify key aspects of your business idea.

Topic	Description
Business Concept	What is your business idea? What products or services do you want to offer and what do you need to do so?
Unique Selling Proposition	What distinguishes your idea? Are there already comparable offers? What do you do differently and how do you stand out from the competition?
Target Audience	Which customer group are you addressing? How do you address these customers? How is the market in this customer segment structured?
Marketing Strategy	What marketing strategies and channels do you use? How much does the advertising cost?
Company Structure	How is the company organised? Which legal form do you choose?
Regulatory Requirements	Do you need permits or technical approvals? What formalities need to be arranged?
Sales Strategy	How do you organise sales? What prices are you aiming for? Do you need a distributor?

**Fig 4:**
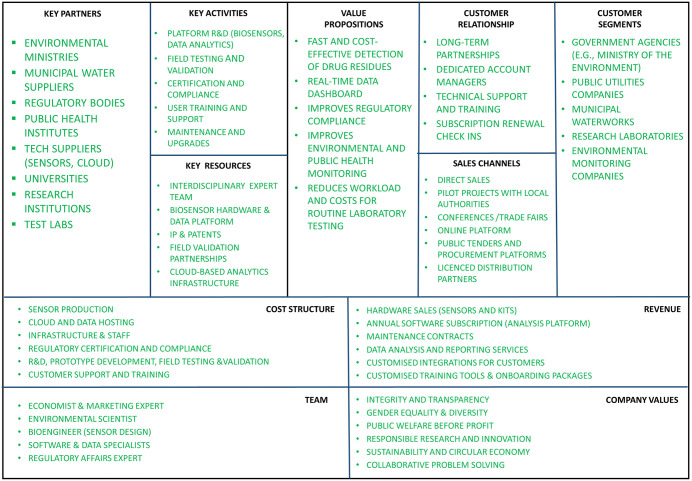
Business Model Canvas—a template. The BMC consists of up to eleven fields, each labelled with the key components of a business model, including two additional elements relevant to early-stage innovation: team and company values. These fields should be filled in and linked together to form a coherent and effective strategy. Social or mission-oriented start-ups can also integrate the social or environmental impact of their solution into this framework [5]. As an example, the fictional screening platform shows how the BMC can be applied in a realistic innovation scenario.

Forty-five minutes to prepare for filling out the BMC should be sufficient. This exercise will facilitate the structuring of the discussed topics so that a more holistic perspective can be obtained. The BMC helps to bring all essential elements of a successful business model into a scalable system. The document comprises up to eleven fields, each labelled with the key components of a business model (Fig 4). The principle is as follows: Ideas are entered into the relevant fields, and computational biology results can be integrated into key areas—for example, the ‘value proposition’ could highlight UV protection efficiency in conjunction with enzyme selection, while ‘key resource’ might include computational pipelines and enzyme libraries. By directly incorporating these computer-assisted insights into the BMC, participants see the concrete connection between laboratory research and market-ready strategy. This visually supported, modular approach allows individual ideas to be compiled and linked until a coherent, marketable business model emerges. The exercise is most effective when conducted by an interdisciplinary group.

Each team will use one BMC to develop their own business model. They will have one hour to finalise their models and prepare a summary for a subsequent presentation. The developed business plan will be presented to potential buyers, investors, collaborators as an “elevator pitch” (see Rule 9)

A structured business plan integrates insights from the IBC process, helping attendees refine their innovation, define a viable business model using the Business Model Canvas, and develop a clear strategy for securing funding, partnerships, and market success.

### Rule 9: Pitch your idea to convince potential investors and future collaborators

Realising a business idea or prototype requires resources such as funding, networks and partnerships. At this stage, your potential stakeholders will provide support for ongoing collaboration and to refine your message to attract investors. The IBC attendees will now actively work on developing an ‘elevator pitch’ aimed at the stakeholders mentioned in **Rule 2**. The elevator pitch is their unique chance to create a strong first impression and convince the stakeholders of their business idea. The team will need guidelines: for example, the elevator pitch should be short and concise—between sixty and ninety seconds, and the following key messages must be included: (i) problem description, (ii) solution approach, and (iii) a call to action. In our example of the new sunscreens, it could look like this: Start by clearly stating the problem that your prototype is targeting—preferably something that resonates with your audience (e.g., pollutants from sunscreen harming marine life). Present the solution—your prototype. Highlight the unique value of your prototype and explain why it stands out from others (e.g., sustainable, biodegradable ingredients with effective UV light filters). Include visualisations of the enzyme’s UV absorption efficiency or prototype design in your presentation to highlight the link between computational biology predictions and product characteristics. Highlighting sustainability and enzyme performance makes the scientific innovation understandable to investors and stakeholders, strengthening both technical credibility and market appeal. Call to action: Conclude with a direct and actionable request, e.g., to arrange a meeting or explore partnership opportunities (e.g., Would you be interested in finding out more?).

The presentation of the various pitches can be turned into an exciting competition. If you have already identified stakeholders from **Rule 2** who would like to support the further development of the business idea, this is a timely opportunity to follow through with this and invite them to the session. Presenting to the IBC attendees themselves can generate valuable feedback as well as engender a sense of camaraderie and healthy competition. You could also consider delivering the presentation to the project partners.

Keep in mind that besides following a business strategy, a convincing pitch could also serve as a springboard for establishing new collaborations or joint applications for project funding.

Make sure that when inviting external stakeholders, **Rule 1** is followed, as this will ensure that the process remains focussed and aligned with your objectives.

An effective elevator pitch equips IBC attendees to secure funding, partnerships and stakeholder support by clearly presenting their problem, innovative solution, and a compelling call to action—turning their business idea into a market-ready opportunity.

### Rule 10: Post-IBC—Feed the momentum

Next, agree with the attendees on the next steps to sustain momentum and continue to drive the progress of the results. Take time to provide constructive feedback to the teams and help them validate or refine their approach. Encourage the teams to share their experiences at the next group meeting, or if it is a project, to report back at the next project meeting or in the project’s newsletter.

This feedback loop ensures that the research remains relevant and adaptable, and that improvements can be made where necessary. Where possible, intensify contact with institutional transfer offices to further develop ideas (use a pitch, see **Rule 9**).

Stakeholder management is also crucial to the process. Maintain contact with the stakeholders you have identified to keep them informed of developments, and nurture relationships that could lead to future collaboration or investment. Their involvement is critical to the sustainable growth of the project. Again, make sure you follow the rules starting from **Rule 1**!

Finally, it is important to encourage everyone involved to carry forward the spirit of adventure and open-mindedness that was fostered during the IBC. This attitude fosters creativity and innovation and ensures that the enthusiasm and momentum generated throughout the process last long after the event is over.

To maintain momentum after the IBC, identify next steps, provide constructive feedback, engage stakeholders and foster collaborative relationships to ensure continuous innovation, adaptability and long-term project growth. This should also include experimental validation of enzyme candidates, formulation stability optimisation, and follow-up discussions with partners identified during Rule 2. To maintain momentum, stakeholders should also be kept informed of progress in the laboratory and the results of market testing so that insights from computational biology continue to drive product development and marketing.

## Conclusion

The IBC serves as an inspirational framework for transforming research ideas into actionable, market-ready solutions. By following the outlined rules—from identifying exploitable results to securing funding and fostering long-term collaboration—attendees can effectively navigate the complex journey of innovation. These steps emphasise a balanced approach that integrates creativity, teamwork, multidisciplinary perspectives and strategic planning, ensuring that ideas are not only generated but also viable in real-world contexts.

Ultimately, the IBC is a dynamic environment that inspires collaboration, encourages bold thinking, and empowers attendees to tackle challenges with a fresh perspective. By maintaining the momentum after the event and continuing to engage with stakeholders, the innovation ignited in the IBC can drive meaningful impact well beyond its initial scope.
